# Complete Genome Sequence of Variant Porcine Epidemic Diarrhea Virus Strain CH/HNZZ47/2016 Isolated from Suckling Piglets in China

**DOI:** 10.1128/genomeA.01744-16

**Published:** 2017-03-02

**Authors:** Xinsheng Liu, Qiaoling Zhang, Yuzhen Fang, Peng Zhou, Yanzhen Lu, Shuai Xiao, Zhaoliang Dong, Yongguang Zhang, Yonglu Wang

**Affiliations:** a State Key Laboratory of Veterinary Etiological Biology, OIE/National Foot and Mouth Disease Reference Laboratory, Key Laboratory of Animal Virology of Ministry of Agriculture, Lanzhou Veterinary Research Institute, Chinese Academy of Agricultural Sciences, Lanzhou, China; b Jiangsu Co-innovation Center for Prevention and Control of Important Animal Infectious Diseases and Zoonoses, Yangzhou, China

## Abstract

Porcine epidemic diarrhea virus (PEDV) could cause an acute and highly contagious enteric disease in swine. Here, we report the complete genome sequence of PEDV strain CH/HNZZ47/2016 isolated from suckling piglets with mild diarrhea in Henan Province, China. It will help understand the molecular and evolutionary characteristics of PEDV in China.

## GENOME ANNOUNCEMENT

Porcine epidemic diarrhea virus (PEDV) is an enveloped, single-stranded, and positive-sense RNA virus in the genus *Alphacoronavirinae* of the family *Coronaviridae* (1, 2). It is the causative agent of porcine epidemic diarrhea (PED), an acute and highly contagious enteric disease characterized by severe enteritis, vomiting, and watery diarrhea in swine (3–5). PED was first reported in China in 1986 and then spread rapidly throughout the country (6). A large-scale outbreak of PED with high morbidity and mortality occurred in swine farms of China in 2010, which led to enormous economic losses (7, 8).

In May 2016, pig diarrheal fecal samples were collected from suckling piglets with mild diarrhea on a swine breeding farm in Henan Province, China. A PEDV strain named CH/HNZZ47/2016 was conformed by reverse transcription-PCR (RT-PCR) targeting the spike gene. To determine the complete genome sequence of the PEDV isolate CH/HNZZ47/2016, 14 sets of primers based on the CV777 strain were designed. The PCR products were cloned into the pMD20-T vector and subsequently sequenced. The complete genome of strain CH/HNZZ47/2016 is 28,023 nucleotide (nt) bases in length, excluding the poly(A) tail. The genomic coding regions are found in the following nucleotide ranges: 5′ untranslated region (UTR), nt 1 to 283; replicase polyprotein, nt 284 to 12592 for 1a and nt 12592 to 20628 for 1b; spike (S), nt 20625 to 24779; open reading frame 3 (ORF3), nt 24779 to 25453; envelope (E), nt 25434 to 25664; membrane (M), nt 25672 to 26352; nucleocapsid (N), nt 26364 to 27689; and 3′ UTR, nt 27690 to 28023.

The complete genome sequence of CH/HNZZ47/2016 shares 99%, 98%, 97%, and 97% nucleotide sequence identity with AH2012, virulent DR13, CH/S, and vaccine strain CV777, respectively. Interestingly, the CH/HNZZ47/2016 strain possesses a remarkable deletion and an insertion site compared to virulent strain CHGD-01 (nt 20815 to 20820 and nt 24222 to 24224) in the S gene, resulting in a continuous 2-amino-acid deletion (^61^GV^62^) and a single-amino-acid insertion (^1197^H). Of note, the deletion site of CH/HNZZ47/2016 is mainly in the S1 domain of the S protein, which is similar to the U.S. S-INDEL strain Iowa106 but shorter. These findings suggested that CH/HNZZ47/2016 is a novel PEDV variant compared with current epidemic virulent strains and indicate that PEDV has shown significant mutations in China. The genome information of strain CH/HNZZ47/2016 will promote a better understanding of the evolutionary characteristics of PEDV in China.

### Accession number(s).

The genome sequence of PEDV strain CH/HNZZ47/2016 has been deposited in GenBank under the accession number KX981440.
